# Micro/Nano Devices for Chemical Analysis

**DOI:** 10.3390/mi7090164

**Published:** 2016-09-09

**Authors:** Manabu Tokeshi, Kiichi Sato

**Affiliations:** 1 Division of Applied Chemistry, Faculty of Engineering, Hokkaido University, Kita 13 Nishi 8, Kita-ku, Sapporo 060-8628, Japan; 2 ImPACT Research Center for Advanced Nanobiodevices, Nagoya University, Furo-cho, Chikusa-ku, Nagoya 464-8603, Japan; 3 Innovative Research Center for Preventive Medical Engineering, Nagoya University, Furo-cho, Chikusa-ku, Nagoya 464-8601, Japan; 4 Institute of Innovation for Future Society, Nagoya University, Furo-cho, Chikusa-ku, Nagoya 464-8601, Japan; 5 Division of Molecular Science, School of Science and Technology, Gunma University, Tenjin-cho, Kiryu, Gunma 376-8515, Japan; kiichi.sato@gunma-u.ac.jp

Since the concept of micro total analysis systems (µ-TAS) has been advocated, various kinds of micro/nano devices have been developed by researchers in many fields, such as in chemistry, chemical engineering, mechanical engineering, electric engineering, biology, and medicine, among others. The analytical techniques for small sample volumes, using the micro/nano devices, heavily impacted the fields of biology, medicine and biotechnology, as well as analytical chemistry. Some applications (DNA analysis, point-of-care testing (POCT), etc.) are already commercially available, and various applications will be put to practical use from now on. In this Special Issue, we focus on chemical and biochemical analyses (analytical and sensing techniques) using the various types of the micro/nano devices, including micro/nanofluidic devices, paper-based devices, digital microfluidics, and biochip (DNA, protein, cell) array. We are also interested in hyphenated devices with other conventional analytical instruments, and the pretreatment devices and components (valve, pump, etc.) for analysis/assay.

The Special Issue of *Micromachines* entitled “Micro/Nano Devices for Chemical Analysis” presents a total of 17 papers, including unique three reviews and two communications. Four papers are related to microfluidic-based sensing techniques, four deal with analysis/assay systems, including a pretreatment system, three focus on the components necessary to build an analysis system, two are on fabrication techniques for 3D structures and 3D microtissues, two focus on paper-based analytical devices, one focuses on the hyphenated device for mass spectroscopy, and the last one shows fundamental research for a droplet injector that might be used as a small volume sample injector.

In sensing technology, it is very important to consider the use of small sample and reagent volumes in micro/nano devices as an advantage. Sarkar et al. [1] show an educational review of electrochemical detection in micro/nanofluidic devices. They also discuss several alternative strategies, aimed at eliminating the reference electrode altogether, in particular, two-electrode electrochemical cells, bipolar electrodes, and chronopotentiometry. Miyamoto et al. [2] propose a plug-based microfluidic system, based on the principle of the light-addressable potentiometric sensor (LAPS). LAPS is a semiconductor-based chemical sensor, which has a free addressability of the measurement point on a sensing surface. They demonstrate the pH sensing of a 400 nL plug. Liu et al. [3] report on frequency domain quasi-optical terahertz (THz) chemical sensing and imaging of liquid samples in microchannels. They demonstrate real-time and label-free chemical sensing and imaging with a broad band width, high spectral resolution, and high spatial resolution. Tao et al. [4] develop a micro-gas detector based on a Fabry-Pérot cavity embedded in a microchannel, and it has a sensitivity of 812.5 nm/refractive index unit (RIU) with a detection limit of 1.2 × 10^−6^ RIU.

There are four papers in this Special Issue describing analysis/assay systems, including a pretreatment system. Gupta and Rezai [5] provide a comprehensive review of microfluidic-based *C. elegans* research. This review focuses on the technological aspects of the progress of microfluidic devices for *C. elegans* research. Phurimsak et al. [6] report a magnetic particle plug-based immunoassay in a microchannel, and apply it to a streptavidin-biotin binding assay, a sandwich assay of C-reactive protein, and a binding assay of progesteronein with a view to achieving competitive ELISA. Navaei et al. [7] study an optimal heater design for a miniaturized gas chromatograph column using numerical simulations. The optimal design is fabricated and evaluated experimentally, and is confirmed to have a good separation performance. Yasui et al. [8] describe 10 µm bead separation in a spiral microchannel using the hydrodynamic separation technique. This technique can be applied to autologous serum eye-drops preparation.

Development of indispensable components, such as valves and pumps, is important to realize real µ-TAS. Yalikun and Tanaka [9] present a fabrication method for the large-scale integration of all-glass valves in a microfluidic device that contains 110 individually controllable diaphragm valve units. Morimoto et al. [10] propose a balloon pump with floating valves to control the discharge flow rates of sample solutions. They demonstrate several microfluidic operations by the integration of the balloon pumps with microfluidic devices. Yalikun et al. [11] report a unique device for three-dimensional micro-rotational flow generation. This device has great potential for fluidic biological applications, such as culturing, stimulating, sorting, and manipulating cells.

Development of new fabrication technologies are always important to the development of this field. Naito et al. [12] present a simple three-dimensional fabrication method, based on soft lithography techniques and laminated object manufacturing. This method is useful, not only for lab-scale rapid prototyping, but also for commercial manufacturing. Che et al. [13] utilize a droplet microfluidic device to fabricate three-dimensional micro-sized tissues (extracellular matrix: ECM) with encapsulated cells. Such 3D microtissues can be applied to studies of cell–ECM interactions and cell–cell communication.

Microfluidic paper-based analytical devices (µPADs) are a relatively new topic and receive a great deal of attention in this field. Busa et al. [14] provide a review of µPADs with specific applications in food and water analysis. µPADs have great potential for practical on-site food and water monitoring. Tenda et al. [15] report a wax-printing-based fabrication method of µPADs for sub-microliter sample analysis. They demonstrate a colorimetric assay of a model protein of 0.8 µL. 

There are two papers covering different aspects of research related to the Special Issue. Mass spectrometry is a powerful tool used to identify unknown compounds within a sample, and is used in a wide range of research fields. Yu et al. [16] report a three-dimensional flow focusing-based microfluidic ionizing source for mass spectrometry that is fabricated using two-layer soft lithography. Kazoe et al. [17] present research on the acceleration of microdroplets (~nL) in the gas phase in a microchannel. While it is still fundamental research, this technique may be applied to a small volume sample injector.

We wish to thank all authors who submitted their papers to this Special Issue. We would also like to acknowledge all the reviewers whose careful and timely reviews ensured the quality of this Special Issue.
